# The Use of the Hydrate of Chloral per Rectum

**Published:** 1878-11-20

**Authors:** W. S. Caldwell


### THE USE OF THE HYDRATE OF CHORAL PER RECTUM.

---

Dr. Starcke, Okerstabsarzt, of Berlin, contributes to the *Berliner Klinische Wochenschift*, No, 33, 1878, an article on the above subject, of which the following is a resume :

"Concerning the continued use (*den chronischen Gebrauch*) of the hydrate of chloral, the minds of the profession at the present time are so divided that I consider it of importance to discuss the subject, especially as the English have lately appointed a commission to study the question of *chloralism* as compared with *morphinism.*

"The piejudice against the frequent use of the remedy in England is very great, prod:.ced, perhaps, by the evil consequences that are said to have followed its administration, especially in the cases of those who were the victims of chronic alcoholism.

"I crnnot deney that I myself had formerly the greatest fear of the use of this hypnotic, produced, perhaps, by a single fatal result that I saw follow the use of a small dose of morphine in an illy-nourished patient, and which caused me to afterwards use sparingly all remedies of this class where the patient was extremely anæmic.

"A few years ago I was taken ill of chronic gastric catarrh, accompanied by an extremely acid condition of the contents of my stomach.

"I became more and more emaciated, lost weight rapidly, and had the general aspect of one suffering from cancer of the stomach.

"In my case, one of the greatest obstacles to a convalesence was a most persistent sleeplessness.

"Although in the evenings I felt the most extreme weariness, and fell asleep as soon as I retired, I would uniformly awake after from one-half to one hour's slumber with my mind full of the events of the day, roll and toss from side to side, perhaps getting a little additional restless sleep towards morning.

"I had now become not only weak in body, but low-spirited, and had entertained the worst forebodings as to the final result in my case.

"I concluded at this juncture to consult a colleague on the subject of the use of the hydrate of chloral.

"Having determined to give the remedy a trial, and wishing to avoid the introducting of anything into the stomach that would increse its irritability, I resolved to use it per enema.

"For this purpose I used an ordinary syringe' with a somewhat long tube attached, injecting 10 grams of a 5 per cent. solution at a temperature of 35°, and after a quarter of an hour I repeated the same quantity, so that in all I used 1 gr^m of the hydrate of chloral.

"A few moments after its introduction I experienced a feeling of bearing down and slight tenesmus, which was soon followed by a snnsation of general comfort, an inward calmness of soul, a convincing evidence of that quiet that was to take the place of my former sleepless nights.

"I soon fell asleep, and had a dreamless repose of five hours, and in fact a comfortable night's rest.

"In this manner I used the hydrate of chloral every night for five months.

"The consequence was that from the day I began its use I began to convalesce.

"From the first my sleep was natural. I awoke with a good appetite, without headache, without stupor, and with a feeling of thankfulness toward an agent that had given me the only quiet sleep that I had had for years."

The doctor says that in spite of its continued use, the effect of the remedy remained the same, although he did not increase the dose, but in fact, as he grew better, he was able to get his night's rest by using only one-half the quantity with which he first began.

By the use of other appropriate treatment for his gastric disease he rapidily grew better, so that during the five months that he took the chloral he gained 30 pounds in weight. He farther says: "I wish to especially emphasize the fact that I had nothing like morphinism produced by its use.

"I had no appetite or desire for the medicine. I lay down every night with the hope that, by the continued improvement in my general health, I would soon be able to sleep spontaneously.

"For the last month I have kept the chloral on my table, as one would carry an unbrella in fine weather, happy in not having to use it.

"Since I have discontinued its use, I have had at no time what could be called a weaning period.

"The use of the hydrate of chloral per rectum has an especial application in diseases of the stomach, on account of its irritating effects upon that organ. I tried twice to take the drug by the mouth, suspended in the yolk of an egg.

"Each time a few moments after I had swallowed the dose, I vomited until I had expelled everything from my stomach, and had none of the soporific effects of the remedy produced.

"One of the principal advantages of using the drug by enema is that it does not undergo tnat decomposition which it always does when taken by the stomach, on account of its coming in contact with the contents of that organ."

The writer dwells upon the necessity of procuring a pure specimen of the drug in order to obtain its good effects, and recommends especially that manufactured by Liebreich, of Berlin.

"I found that all the disagreeable sensations about the rectum produced by the injection of the remedy were coufinded to the external margin of the anus, and that all that was necessary to avoid this is to use an instrument with a long tube attached, and exercise some caution to prevent the fluid coming in contact with the external sphincter.

"Care should be taken to have your remedy well desolved, and it shold be used at a temperature near that of the human body.

"The size of the dose necessary to produce a hypotic effect is so small that it cannot be dangerous."

The doctor claims to have used this drug largely in his own practice, as above recommended, and with uniformly the most happy results. In consequence, also, as he asserts, of a growing fear of the use of morphine hypodermically, by the profession at large, in Germany, he thinks the use of the hydrate of chloral is destined to obtain a large field of usefulness.

"In a large number of old people who constantly trouble the physician on account of their sleeplessness, I have found the use of this remedy in the manner aboved described to be of especial benefit, but I should limit the dose given to 1 gram.

"I believe that this dose of the drug used in this way, can have no worse effect than a simple glass of strong wine, which is so often prescribed for this class of patients.

"In conclusion, I would say that in my own case, as well as in the cases that have occurred in my private practice, I have seen no injurious consequences either to the nervous or digestive systems, follow as a result of the effect of the medicine, even when the same was continued for months."

In the minute directions that Dr. Starcke gives for the use per enema of of the hydrate of chloral, I think he has omitted one important point, and that is, that the rectum should be well cleared by an injection of warm water before the remedy is used, if we are going to be sure that we will have none of the obstacles to the complete action of the drug that he claims we are so likely to have when it is given by the mouth.—*W. S. Caldwell, in Chicago Medical Examiner.*

